# Calcium Imaging Characterize the Neurobiological Effect of Terahertz Radiation in Zebrafish Larvae

**DOI:** 10.3390/s23187689

**Published:** 2023-09-06

**Authors:** Xin Song, Haibin Li, Xiuyun Liu, Meijun Pang, Yuye Wang

**Affiliations:** 1 Academy of Medical Engineering and Translational Medicine, Tianjin University, Tianjin 300072, China; songxin_20@tju.edu.cn (X.S.); xiuyun_liu@tju.edu.cn (X.L.); 2 School of Precision Instruments and Optoelectronics Engineering, Tianjin University, Tianjin 300072, China; haibin_li@tju.edu.cn

**Keywords:** terahertz radiation, zebrafish, neural calcium imaging, neurobiological effect

## Abstract

(1) Objective: To explore the neurobiological effects of terahertz (THz) radiation on zebrafish larvae using calcium (Ca^2+^) imaging technology. (2) Methods: Zebrafish larvae at 7 days post fertilization (dpf) were exposed to THz radiation for 10 or 20 min; the frequency was 2.52 THz and the amplitude 50 mW/cm^2^. The behavioral experiments, neural Ca^2+^ imaging, and quantitative polymerase chain reaction (qPCR) of the dopamine-related genes were conducted following the irradiation. (3) Results: Compared with the control group, the behavioral experiments demonstrated that THz radiation significantly increased the distance travelled and speed of zebrafish larvae. In addition, the maximum acceleration and motion frequency were elevated in the 20 min radiation group. The neural Ca^2+^ imaging results indicated a substantial increase in zebrafish neuronal activity. qPCR experiments revealed a significant upregulation of dopamine-related genes, such as *drd2b*, *drd4a*, *slc6a3* and *th*. (4) Conclusion: THz radiation (2.52 THz, 50 mW/cm^2^, 20 min) upregulated dopamine-related genes and significantly enhanced neuronal excitability, and the neurobiological effect of THz radiation can be visualized using neural Ca^2+^ imaging in vivo.

## 1. Introduction

A terahertz (THz) wave refers to electromagnetic waves with frequencies ranging from 0.1 THz (100 GHz) to 10 THz and wavelengths from 0.03 mm to 3 mm, which shows both optical particle-like and electric wave-like characteristics. It has opened up new avenues for analyzing, identifying, and regulating the structure and properties of biological macromolecules. Furthermore, THz waves can interact with biomolecules through mechanisms such as vibration, rotation, weak hydrogen bonding, or van der Waals forces. These low-energy motions are closely related to the biomolecules’ structural and functional changes [1]. After decades of development in THz technologies, the applicability of these technologies has been demonstrated in a wide range of fields, including safety inspection, materials science, biomedical diagnosis, communication and environmental science [2]. To enable a wider spread of the practical applications of THz, it is crucial to ensure the safety of THz radiation for human health [3].

In recent years, increasing evidence has revealed the effects of THz radiation on the structure and function of the nervous system. At the cellular level, THz radiation at 0.72 THz with the power intensity of 10–20 mW/cm^2^ caused isolated neuronal cell adhesion and synaptic structure changes after 60 min exposure, while high power THz radiation at 3.68 THz resulted in the structural changes of the cell membrane, axon and growth cone of the same neurons [4]. THz radiation at a frequency of 3.1 THz enhanced excitatory synaptic transmission and discharge of cortical neurons [5]. Furthermore, when exposed to THz radiation at 3.1 THz for 2 days (3 h a day), the proliferation of oligodendrocyte progenitor cells (OPCs) was inhibited, while differentiation and myelination were promoted. These findings indicate that THz radiation regulates the function of different neurons, and the sensitivity of different neurons to THz waves varies. At the animal level, the THz wave affects the central nervous system (CNS) and the behavior of animals. For example, THz radiation at 150 GHz and 0.2 mW/cm^2^ for 30 min effectively suppressed the routine activities and exploratory behavior of rats [6]. In addition, THz radiation at 150 GHz and 3 mW/cm^2^ for 60 min enhanced platelet aggregation and induced depressive symptoms [7]. Additionally, THz radiation at 3.6 THz and 23.6 mW/cm^2^ for 30 min increased the anxiety levels in mice [8]. Rats subjected to 144 GHz for five days exhibited anxiety symptoms, decreased appetite and reduced sleep duration. Therefore, animals exhibit different behaviors following THz radiation at different frequencies. However, the complex molecular mechanisms of THz radiation on the nervous system require further investigation. Furthermore, it is worth noting that there needs to be more in vivo visualization research on the neurobiological effects of THz radiation.

Zebrafish, a vital animal model, possess strong reproductive ability, transparent embryos, a small size, low neurological complexity, and high genetic and organ similarities to humans. This model has a significant research value in the fields of development, pathology and toxicology and has been widely used in bioimaging and neuroscience research [9,10,11]. The tiny and transparent brain of the zebrafish larvae is particularly suitable for the optical monitoring and manipulation of whole-brain neurons at cell resolution [12,13]. With the advancements in genetics and gene-editing technology, researchers have developed various types of calcium indicators, with GCaMP6 being widely used for in vivo calcium imaging, due to its exceptional sensitivity [14,15,16,17].

In the current study, we investigated the neurobiological effects of THz radiation in vivo. In addition to conducting behavioral experiments, we employed neural activity imaging techniques to investigate the effects of THz radiation on Tg (HuC: GCaMP6S) transgenic lines of zebrafish. By utilizing in vivo visualization imaging, we were able to efficiently observe and analyze the neurobiological effects induced by THz radiation. Compared with the control group or the masked group, we observed a significant enhancement of whole-brain neural-calcium transmission, especially in the telencephalon, after 20 min of irradiation. We also tested dopamine-related genes with qPCR, and the expression levels of *drd2b*, *drd4a*, *slc6a3* and *th* were significantly upregulated in the 20 min radiation group. These results provide evidence for the neurobiological effects of THz radiation in vivo and confirm the feasibility of nerve visualization imaging for evaluating THz effects. Moreover, our findings contribute to THz’s theoretical basis in neuroscience and clinical applications.

## 2. Materials and Methods

### 2.1. Zebrafish Husbandry and Embryo Collection

Unless otherwise stated, wild-type (TU strain) zebrafish were used for the experiments. Zebrafish Tg (HuC: GCaMP6S) line was employed to evaluate the excitability of neuron activities [18]. Adult zebrafish were maintained at 28 ± 0.5 °C under a constant 14 h light/10 h dark cycle photoperiod. The Tg (HuC: GCaMP6S) line was crossed with TU at the ratio of 2:1 (female: male) and embryos were collected the next morning. The embryos were placed in 28 ± 0.5 °C incubators, and the E3 medium (5 mM NaCl, 0.17 mM KCl, 0.4 mM CaCl_2_, and 0.33 mM MgSO_4_) was changed daily. All the animal procedures were approved by the Animal Ethics Committee of Tianjin University (protocol code TJUE-2023-010).

### 2.2. THz Radiation and Laser Confocal Imaging

**Figure 1:**
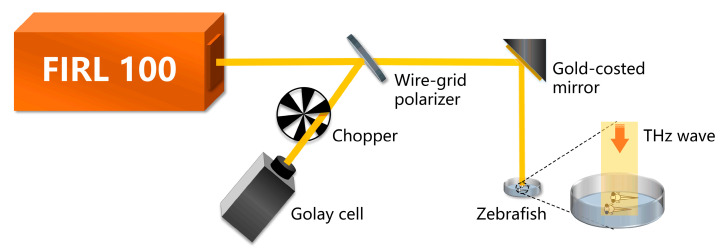
Schematic diagram of the THz radiation experimental set-up. Zebrafish were placed in a sample pool and the THz wave at 2.52 THz was irradiated onto the surface of the sample pool.

Zebrafish larvae were placed in the sample pool at 7 days post-fertilization (dpf). Then the zebrafish larvae were fixed in a prone position with a 0.4% low melting point agarose thin layer. The control group was not subjected to THz radiation. The masked group was covered with tin foil, which prevented the transmission of THz waves. The radiation group was directly radiated by the THz wave. For this experiment, as shown in Figure 1, an optically pumped THz gas laser (FIRL 100, Edinburgh Instruments Ltd., Livingston, UK) with tunable continuous-wave THz output was employed as the THz emission source. A frequency of 2.52 THz was selected for this study due to two key factors. Firstly, the chosen frequency aligns with the higher output power capabilities of the THz laser, potentially reaching up to 150 mW. This elevated output power enhances the efficiency of experimental procedures and bolsters signal-to-noise ratios. Secondly, the operational stability of the THz source is significantly improved when operating at 2.52 THz. This enhanced stability is imperative for the precise investigation of neural activity through the utilization of THz radiation. The THz wave was divided into two beams by a wire-grid beam splitter (Micromesh Instruments, Inc., Lorton, USA), where one beam served to irradiate zebrafish and the other was used as a reference to monitor the power fluctuation. The reference light was detected using the Golay cell detector (GC-1P, Tydex Ltd., Peterburg, Russia). The emitted laser beam exhibited a spot size of 10 mm in diameter. It can effectively cover all zebrafish larvae within the sample pool, considering their body length of approximately 3–5 mm. Furthermore, it is noteworthy that the FIRL 100 laser offers a power stability of around 2.03% for THz wave emission over a 60-min period. These considerations collectively contribute to maintaining the parameters’ stability during the experiment. To avoid the influence of E3 on irradiation results, excess E3 on the surface was wiped away and then the sample pool was positioned at the outlet of the THz source. Additionally, to ensure the activity of zebrafish, the sample pool was taken down and E3 was added after irradiation. Then, the sample pool was placed under the laser confocal fluorescence microscope (Nikon A1R) for three-dimensional head imaging.

### 2.3. Temperature Measurements 

In the experiment, we employed an electronic digital display thermometer with a highly sensitive probe (featuring a detection sensitivity of 0.1 °C and a refresh rate of 2 s) to measure temperatures before and after THz irradiation. To further elaborate on the procedure, we placed the sample pool containing fixed zebrafish larvae in the laboratory environment until their temperature equilibrated with the room temperature. Subsequently, the sample pool was positioned at the outlet of the THz source. Temperature measurements were performed by inserting the probe into the agarose surrounding the zebrafish larvae, and readings were taken when the temperature values stabilized. Since zebrafish are poikilothermic organisms, their body temperature varies with changes in the environment’s temperature. As a result, the temperature of the measured agarose can be considered equivalent to that of the zebrafish larvae. To ensure accuracy, we conducted three independent measurements for each experimental group and calculated the average value. 

### 2.4. Zebrafish Behavior Experiment

Zebrafish larvae were placed into 48-well plates (one embryo per well) randomly, and one-hour motion trials were recorded with 10 min/10 min light/dark cycle after adaptation for 20 min by DanioVision (Noldus). The total swimming distance, swimming speed, and moving cumulative duration were analyzed.

### 2.5. qPCR Experiment

Zebrafish larvae were collected and placed in the RNase-free EP tube, E3 was wiped away, and TRIzol (Gene-Protein Link, CAT: G01E05M) added. Total mRNA was extracted and reverse-transcribed into cDNA (Yeasen, CAT:11141ES60). Hieff qPCR SYBR Green Master Mix (Yeasen, CAT:11201ES03) and gene-specific primers were used for qPCR. Bio-Rad CFX96 real-time system (Hercules, CA, USA) was used for detection and analysis. The mRNA relative expression level of the target gene was calculated by 2^−ΔΔCt^. The primer sequence used in this study is shown in the Supplemental Information Table S1.

### 2.6. Statistical Analysis

All data are presented as the mean ± standard error of the mean (SEM) of experiments. The significance was determined by Student’s *t*-test in GraphPad Prism 8. Significant differences were identified when the *p*-value was lower than 0.05. (* *p* < 0.05, ** *p* < 0.01, and *** *p* < 0.001).

## 3. Results

### 3.1. THz Radiation Promotes the Behavior of Zebrafish Larvae

**Figure 2:**
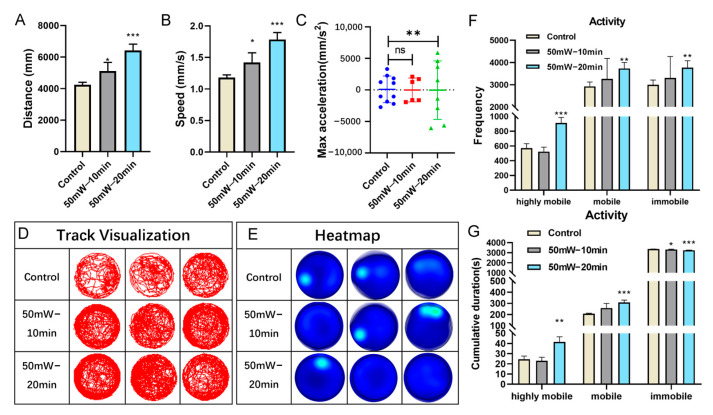
The effects of THz radiation on swimming behaviors of zebrafish larvae. (**A**–**C**) The total swimming distance (**A**), swimming speed (**B**), and max acceleration (**C**) of zebrafish larvae at 7dpf. (**D**,**E**) The track visualization (**D**) and the heatmap (**E**) for 1 h. (**F**,**G**) The frequency (**F**) and the cumulative duration (**G**) of three states of zebrafish larvae. Three independent experiments were performed, with 8 subsamples per dose per replication. These data are presented as the mean ± SEM, * *p* < 0.05, ** *p* < 0.01 and *** *p* < 0.001 vs. control.

Behavior is an essential indicator for evaluating neural function. To examine the effect of THz radiation, the current study conducted a behavior assay of zebrafish larvae at 7 dpf. Compared with the control group, the zebrafish larvae in the 10-min or 20-min radiation group (2.52 THz, 50 mW/cm^2^) exhibited a significant increase in both total swimming distance and speed (Figure 2A,B). Interestingly, the maximum acceleration in the 20 min radiation group showed a substantial increase (Figure 2C). Figure 2D,E show the swimming track and the heatmap of the zebrafish larvae. Based on the swimming speed, we divided the motion state into three categories: highly mobile state, mobile state, and immobile state. A significant increase in the frequency observed in the 20 min radiation group was detected for all three stages (Figure 2F). Similarly, the cumulative duration in both the highly mobile state and the mobile state increased. At the same time, it was decreased in the immobile state (Figure 2G). In addition, the cumulative duration in the immobile state decreased in the 10 min radiation group, while no significant changes were observed in both the highly mobile state and mobile state. Taken together, these results revealed the effect of THz radiation (2.52 THz, 50 mW/cm^2^, 20 min) in promoting the swimming behaviors of zebrafish larvae.

### 3.2. THz Radiation Promotes the Neural Activities of Zebrafish Larvae

**Figure 3:**
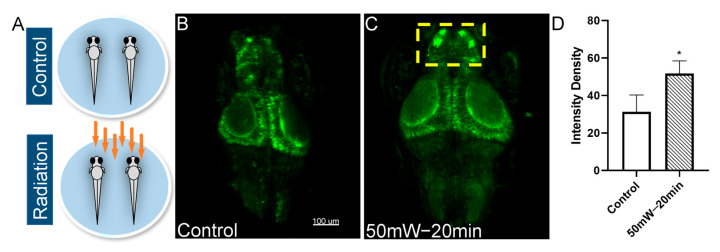
The effect of THz radiation on neural Ca^2+^ activities of zebrafish larvae. (**A**) Schematic representation of the THz radiation. (**B**,**C**) Representative images of neural Ca^2+^ in the control and the radiation group at 7dpf. (**D**) Fluorescence intensity of neural Ca^2+^. Scale bar, 100 µm. N = 5. These data are presented as the mean ± SEM, * *p* < 0.05 vs. control.

Neuronal Ca^2+^ imaging has become an essential method in modern neuroscience. In this study, we chose the zebrafish Tg (HuC: GCaMP6S) line, which exhibits a pan-neuronal expression of GCaMP6f, to evaluate the neurobiological effects of THz radiation on neural activities. The experimental design is shown in Figure 3A. Compared with the control group, the neural Ca^2+^ fluorescence intensity in the radiation group was significantly enhanced, especially in the telencephalon region (Figure 3B–D). The results indicated an enhancement of neuronal excitability in the zebrafish brain due to THz radiation (2.52 THz, 50 mW/cm^2^, 20 min).

**Figure 4:**
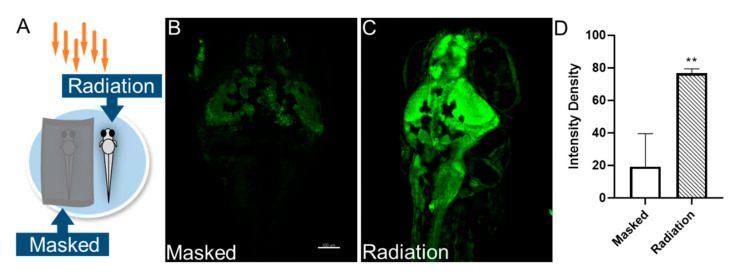
The effect of THz radiation on neural Ca^2+^ activities of zebrafish larvae. (**A**) Schematic representation of the THz radiation. (**B**,**C**) Representative images of the neural Ca^2+^ in the masked and the radiation group at 7dpf. (**D**) Fluorescence intensity of neural Ca^2+^. Scale bar, 100 µm. N = 5. These data are presented as the mean ± SEM, ** *p* < 0.01 vs. the masked group.

The biological changes caused by THz irradiation include thermal effects and non-thermal effects (also called biological effects). Studies have also shown that the THz wave could induce changes in the temperature of biological substances and increase the level of heat shock proteins in mammals [19]. Therefore, to further investigate whether the non-thermal effect or thermal effect caused the neural activity changes in zebrafish, we recorded the temperature of the zebrafish before and after irradiation. We observed a temperature increase of approximately 0.1 °C (ranging from 0 to 0.2 °C in the six groups, Table 1). Furthermore, we updated the experimental design (Figure 4A). The masked group was covered with tin foil, which prevented the transmission of THz waves. It should be noted that they may experience temperature changes due to localized heat buildup. Therefore, this design aims to isolate the non-thermal effects of THz radiation, and only subjected them to thermal effects. Consistent with the previous results, THz radiation (2.52 THz, 50 mW/cm^2^, 20 min) enhanced the neural Ca^2+^ fluorescence intensity in the zebrafish brain (Figure 4B–D). Together, these experiments demonstrated that THz radiation enhanced the neuronal excitability of zebrafish brains primarily through neurobiological effects rather than thermal effects.

### 3.3. THz Radiation Promotes the Dopamine-Related Gene Expression of Zebrafish Larvae

**Figure 5:**
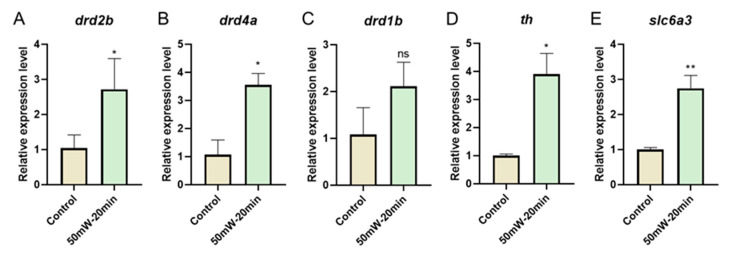
The effects of THz radiation on mRNA expression of the dopamine-related gene. (**A**–**C**) The mRNA levels of dopamine receptor genes *drd2b* (**A**), *drd4a* (**B**) and *drd1b* (**C**). (**D**,**E**) The mRNA levels of dopamine synthesis gene *th* (**D**) and dopamine transport gene *slc6a3* (**E**). These data are presented as the mean ± SEM, * *p* < 0.05, ** *p* < 0.01 vs. control.

To understand the molecular underpinnings of the observed changes in neuronal excitability following THz irradiation, we compared the gene expression between the control and THz radiation groups. The dopaminergic system plays an important role in regulating behavioral effects, as dopamine is activated and released when the nervous system is excited [20]. Therefore, we examined the dopamine-related gene expression after irradiation. qPCR revealed a significant upregulation in the *dopamine receptor genes, dopamine receptor D2b* (*drd2b*) and *dopamine receptor D4a* (*drd4a*) in the THz radiation group (2.52 THz, 50 mW/cm^2^, 20 min) (Figure 5A,B), as well as the dopamine synthesis gene *tyrosine hydroxylase* (*th*) and dopamine transport gene *solute carrier family 6 member 3* (*slc6a3*) (Figure 5D,E). However, we found no significant change in the expression of dopamine receptor D1b (drd1b) (Figure 5C). Together, these observations suggest that THz radiation (2.52 THz, 50 mW/cm^2^, 20 min) can enhance neuronal excitability in vivo by promoting the process of dopamine synthesis and transport.

## 4. Discussion

In recent years, there has been increasing evidence revealing the effects of THz radiation on the structure and function of the nervous system [21]. However, the neurobiological effects of THz still need to be better understood due to the different experimental designs (radiation conditions, in vitro or in vivo models, etc.) and detection methods used. In this study, we employed GCaMP6S-expressing zebrafish to assess the neurobiological effect of THz radiation on neural activities in vivo. We found that THz radiation (2.52 THz, 50 mW/cm^2^, 20 min) significantly enhanced neuronal excitability in zebrafish. Furthermore, we demonstrated that THz radiation also promoted the expression of dopamine receptor genes such as *drd2b* and *drd4a*, as well as the levels of the dopamine transport gene *slc6a3* and dopamine synthesis gene *th*. In addition, it increased the swimming behavior of zebrafish larvae. Consistent with the molecular level results, there were significant increases in the total swimming distance, speed, and max. acceleration. Therefore, THz radiation with a specific frequency and duration could act as a neurostimulator to enhance neuronal activities in vivo and promote motor behavior by increasing the expression of dopamine-related genes.

In the study, the temperature of the zebrafish larvae increased by approximately 0.1 °C after irradiation. However, Wilmink et al. found that the temperature of a human dermal fibroblast sample increased approximately 3 °C after irradiation by a 2.52 THz source with an intensity of 84.8 mW/cm^2^ for 20 min [19]. It is speculated that the dielectric properties play a pivotal role in determining the response of biological tissues to THz irradiation. The dielectric properties of biological tissues, including permittivity (ε) and conductivity (σ), significantly impact their interaction with THz radiation. However, these properties can vary widely between different tissues and species. Zebrafish larvae and human dermal fibroblasts are distinct biological entities with diverse cellular compositions, sizes, and physiological characteristics. These differences are likely to result in varying dielectric responses to THz radiation due to factors such as water content, lipid composition, cellular density, and intracellular structures. Unfortunately, specific dielectric-property data for these tissues in the THz frequency range may not be readily available. This underscores the need for further investigation into the dielectric properties of zebrafish larvae and human dermal fibroblasts under THz irradiation conditions to better understand their responses and the observed temperature differences between the studies.

Our results indicated that THz radiation primarily promotes the behavior of animals through nonthermal effects [6,8]. In this study, the breeding environment temperature of the zebrafish and the laboratory temperature of THz irradiation was 28 ± 0.5 °C and 19.5 ± 0.5 °C, respectively. However, the temperature of the zebrafish larvae had a negligible change by approximately 0.1 °C after irradiation. Therefore, it is reasonable to assume the observed increases in neural Ca^2+^ activities are mainly caused by the neurobiological effect rather than the thermal effect. In addition, Bo et al. conducted a simulation experiment of temperature change in nerve cells, proving that the effect of THz irradiation on opening pressure-controlled calcium-ion channels and thereby increasing the intracellular Ca^2+^ concentration was a nonthermal effect [22]. Furthermore, our findings suggest that THz radiation promotes the swimming behavior of zebrafish larvae by increasing the expression of dopamine-related genes. Similar to these results, previous studies revealed that THz radiation could induce structural and functional changes in the neurons by altering the protein structure and gene expression, thereby influencing animal behavior [23,24]. It should be noted that the effects of THz radiation on animal behavior may vary depending on the radiation frequency and duration [6]. 

When studying neuronal activity, neural Ca^2+^ signals are essential neurotransmitters in neurons widely used to characterize neuronal activity. The intracellular Ca^2+^ signal exhibits dynamic variations during the neuronal action potential and can increase rapidly by 10–100 times [25]. Therefore, neural Ca^2+^ imaging has been extensively employed in detecting neuronal development and function, enabling direct visualization of dynamic changes in living nerve cells and tissues [26]. Additionally, the patch clamp technique is a classic way to monitor the membrane potential and assess the changes in brain function at different spatial scales. In recent years, various voltage sensors, including gene-coded voltage sensors and photovoltage sensors, have been reported for recording neuronal membrane potential [27,28]. In addition, neurotransmitters serve as important messengers for information transmission between synapses and play a key role in mediating the information transmission of neurons. When combined with imaging systems, neurotransmitter fluorescence probe technology allows the real-time detection of changes in neurotransmitter concentrations in vivo, offering high sensitivity, strong molecular specificity, and high spatial resolution. Common neurotransmitters include dopamine, acetylcholine, and norepinephrine, which are critical for regulating learning, memory, sleep, movement, and other processes [29,30,31,32]. In recent years, Li Yulong et al. developed the Tg (elval3: DA1m) zebrafish line based on a dopamine fluorescence probe [33]. In addition, they also synthesized acetylcholine fluorescence probes and norepinephrine probes for in vivo neuroimaging of zebrafish [34,35]. Using these methods to detect the effects of THz radiation on neuronal activity in vivo models represents another critical research direction for the future. 

THz waves have broad potential applications in improving and treating nervous system diseases. The nervous system functions on bioelectricity and is more susceptible to THz waves. The dopaminergic system controls various important central nervous system (CNS) functions, such as motor control, reward behavior, and cognitive levels [36,37,38]. In this study, our observations indicated a significant upregulation in the expression of dopamine receptor genes *drd2b* and *drd4a*, dopamine synthesis gene *th,* and dopamine transport gene *slc6a3* after THz irradiation. In addition, other studies have shown that THz waves can reduce the dissociation of dopamine drugs and decrease the side effects of psychotropic drug treatment [39]. Together, the results suggested that THz waves may regulate dopamine by interacting with related biomolecules. 

Besides THz waves, mid-infrared waves and microwaves all belong to electromagnetic waves. Mid-infrared waves are also widely used to regulate biological neural activity because of their excellent penetration into biological tissue. Research has shown that mid-infrared stimulation (MIRS) could accelerate the repolarization process of neuronal signals by reversible resonance in the carbonyl groups of the K+ channel [40,41]. A mid-infrared modulation (MIM) stimulation was also reported and it was suggested that it significantly enhanced the electrical activity of neurons, thereby displaying a faster learning rate in mice [42]. However, there are few studies on microwave irradiation’s positive regulation of neuronal activity. Studies have found that microwave radiation damages the synaptic plasticity of neurons and activates neuron autophagy, thereby impairing learning and memory [43,44,45,46]. In summary, mid-infrared or THz waves with the appropriate frequency and intensity can enhance neuronal function. However, it has been suggested that microwave stimulation can cause extreme damage to the nervous system.

Whole-brain imaging of neurons is an effective method for deciphering brain function and neuronal responses. In this experiment, we performed whole-brain neural Ca^2+^ imaging on zebrafish larvae, representing the first-time in vivo neuroimaging was used to investigate the neurobiological effects of THz radiation. However, this study has two limitations. Firstly, it was unable to capture real-time neuronal activity during THz radiation. Secondly, the response pathways of different neurons to THz radiation could not be determined. In recent years, some researchers have combined light stimulation with the microscope using targeted photogenetic technology and integrated the behavior monitoring module into the system [47]. This method enables the examination of the response of the entire neural circuitry to stimulation by combining neural stimulation with neuronal imaging and tail activity. Furthermore, light-sheet fluorescence microscopy (LSFM) offers high spatiotemporal resolution, low photobleaching and low phototoxicity, making it suitable for long-term, live 3D imaging. LSFM has been widely employed in brain neuronal imaging and cerebrovascular imaging in zebrafish [48,49]. In the future, applying this method could allow for real-time monitoring of neuronal responses to THz radiation, thereby facilitating a deeper analysis of the neurobiological effects of THz radiation and providing a more solid theoretical foundation for the application of THz in neuroscience and the treatment of neurological diseases.

## Figures and Tables

**Table 1:** Temperature changes before and after THz radiation.

Group	Temp before Irradiation (°C)	Temp after Irradiation (°C)	Difference Value
1	19.7	19.8	0.1
2	19.7	19.9	0.2
3	19.7	19.9	0.2
4	19.7	19.8	0.1
5	19.7	19.8	0.1
6	19.7	19.7	0

## Data Availability

The authors confirm the availability of all data generated or analyzed in this manuscript, and the data used to support the findings of this study are available from the corresponding author or first author upon request. If you have any questions, please context meijun.pang@tju.edu.cn.

## Supplementary Materials

The following supporting information can be downloaded at: https://www.mdpi.com/article/10.3390/s23187689/s1, Table S1: Genes and primers.
