# Supplementary material for: Calcium Imaging Characterize the Neurobiological Effect of Terahertz Radiation in Zebrafish Larvae
Source: Sensors (Basel). 2023 Sep 6;23(18):7689. doi: 10.3390/s23187689 (PMC10537331; doi:10.3390/s23187689)
Supplement: Supplementary file 1 [file sensors-23-07689-s001.zip › sensors-2532393-supplementary.pdf]

Table S1. Genes and primers.

Gene	F Primer	R Primer
drd1b	ACTGCATGGTTCCTTTTTGC	GGATTTGTGCTGTCCGTTTT
drd2b	GTCCTGAACGTGACAGAAGAG	CTCACCAACAACCTCCAGATAG
drd4a	GACCGTATGTGATGGACTGATG	CGAACAGACGGAGGAGTAGATA
th	ATCAGGATCACCCAGGATTTAC	CCAAGAGATGCCAAACCAATAC
slc6a3	AGACATCTGGGAAGGTGGTG	ACCTGAGCATCATACAGGCG
gadph	ATCATCTCTGCCCCAAGTGC	AGTCAGTGGACACAACCTGG
